# Advanced Imaging Techniques for Early in‐Vivo Discrimination of Fibrofolliculomas in Birt‐Hogg‐Dubé Syndrome

**DOI:** 10.1111/srt.70381

**Published:** 2026-09-12

**Authors:** Maximilian Deußing, Daniela Hartmann, Pontus Mertsch, Charlotte Gust, Michael J. Flaig, Marlene Reithmair, Julia Hoefele, Elke C. Sattler

**Affiliations:** ^1^ Department of Dermatology and Allergy LMU University Hospital, LMU Munich Munich Germany; ^2^ Department of Dermatology and Allergy Munich Municipal Hospital Munich Germany; ^3^ Department of Medicine V University Hospital, LMU Munich München Germany; ^4^ Institute of Human Genetics LMU University Hospital, LMU Munich Munich Germany

**Keywords:** histopathology, line‐field confocal OCT, non‐invasive imaging, rare skin diseases

## Abstract

**Background:**

Birt‐Hogg‐Dubé syndrome (BHDS) is a rare genetic tumor syndrome characterized by cutaneous fibrofolliculoma and trichodiscomas, lung bullae with an elevated risk of spontaneous pneumothorax and renal cell cancer.

**Objectives:**

Due to rareness of the disease, clinical symptoms and cutaneous manifestations are often misjudged, resulting in delayed medical diagnosis and considerable health risks for patients and their families. Accurate discrimination of fibrofolliculomas from other non‐malignant skin tumors can be crucial for early diagnosis and appropriate management of BHDS. In addition, they may clinically overlap with adnexal or follicular papules seen in other rare genodermatoses, including facial angiofibromas in tuberous sclerosis complex, trichilemmomas in Cowden syndrome/PTEN hamartoma tumor syndrome, and trichoepitheliomas in CYLD cutaneous syndrome.

**Methods:**

In this study, skin lesions from 15 BHDS patients with genetic confirmed disease‐causing variants in *FLCN* underwent reflectance confocal microscopy (RCM) and Line‐Field Confocal Optical Coherence Tomography (LC‐OCT) to systematically analyse the morphologic and microstructural features of fibrofolliculomas.

**Results:**

In RCM a dilated follicular unit was seen in 12/15 cases (80%), while in LC‐OCT this feature was seen in 13/15 (87%). The characteristic round, onion‐like shaped hyperreflective structures with a well‐demarcated perifollicular halo, corresponding to the histopathological features of branching chords and strands of epithelial cells was seen in all cases (RCM and LC‐OCT 100%), aiding clinicians in identifying fibrofolliculomas.

**Conclusion:**

Advanced imaging techniques provide a non‐invasive approach for early diagnosis of fibrofolliculomas. Therefore, RCM and especially LC‐OCT may be promising tools for enhancing the diagnostic precision and sufficient clinical management of Birt‐Hogg‐Dubé syndrome.

## 1 Introduction

Birt‐Hogg‐Dubé syndrome (BHDS), a rare, hereditary tumor syndrome, is characterized by a spectrum of clinical manifestations, primarily including cutaneous fibrofolliculomas and trichodiscomas, renal tumors and pulmonary cysts [1, 2, 3]. It is caused by disease‐causing variants (pathogenic and likely pathogenic variants) in the *FLCN* gene, leading to dysregulation in cell growth and proliferation [4, 5, 6]. Fibrofolliculomas, the hallmark dermatological feature of BHDS, typically appear as small, skin‐colored or slightly whitish‐yellowish papules, primarily distributed on the face, retroauricular region, neck and upper trunk, usually develop between the age of 20 and 40 years and affect approximately up to 80% of individuals with BHDS over the age of 40 years [7]. While fibrofolliculomas are generally benign, they can serve as indicators being crucial for early diagnosis and effective management of BHDS, as the syndrome is associated with an increased risk of renal cell carcinoma and pneumothorax [8].

Importantly, the presence of multiple histologically confirmed fibrofolliculomas or trichodiscomas is considered one of the major clinical diagnostic criteria for BHDS. Before genetic testing for variants in *FLCN* became available, the identification of these lesions was central to diagnosing the syndrome. Even today, in approximately 4%–5% of clinically suspected BHDS cases, no disease‐causing variant in *FLCN* can be detected despite comprehensive testing [9]. In such cases, proof of fibrofolliculomas remains critical for establishing the diagnosis, guiding further systemic evaluation and family screening [9, 10].

Diagnosing fibrofolliculomas and distinguishing them from other non‐malignant skin tumors pose significant challenges due to their variable clinical presentation, rarity and overlap with other skin lesions. A major diagnostic hurdle lies in the fact that BHDS is still underrecognized in dermatologic practice [11]. Many clinicians may never have encountered a case, as fibrofolliculomas are often misdiagnosed as more common conditions such as acne, rosacea or seborrheic skin changes — differentials that dominate routine dermatologic evaluations. These frequent misclassifications can delay the correct diagnosis and, consequently, the detection of potentially life‐threatening systemic manifestations such as renal tumors or pneumothorax [3].

Traditional diagnostic tools, including clinical examination and dermoscopy often lack the requisite resolution and specificity needed to precisely differentiate fibrofolliculomas from these more prevalent but unrelated conditions. [12].

As a result, up to now invasive histopathological analysis is the diagnostic gold standard. Nevertheless, it is time consuming and due to the main location on the face, biopsies can result in unfavorable scars in cosmetically sensitive regions [13]. Consequently, there is a pressing need for advanced imaging techniques that can provide detailed but non‐invasive insights beneath the skin into the microstructural and cellular characteristics of fibrofolliculomas.

In recent years, Line‐Field Confocal Optical Coherence Tomography (LC‐OCT) and Reflectance Confocal Microscopy (RCM) have emerged as promising non‐invasive imaging modalities in dermatology, enabling real‐time visualization of skin microstructures up to cellular resolution [14, 15]. These techniques offer the advantage of capturing high‐resolution images of tissue architecture, cellular morphology and microstructural features in vivo, thus providing valuable information for accurate diagnosis and monitoring of cutaneous lesions. Leveraging the potential of LC‐OCT and RCM in the context of BHDS may offer a novel and powerful approach to differentiate fibrofolliculomas from other skin tumors, thereby enhancing the clinical management and prognosis of individuals affected by this syndrome.

This study aims to explore the utility of LC‐OCT and RCM in characterizing fibrofolliculomas, contributing to the advancement of diagnostic and therapeutic strategies for BHDS.

## 2 Materials and Methods

A total of 15 BHDS patients with a genetically confirmed diagnosis of BHDS (disease‐causing variants in *FLCN*), which had previously undergone genetic testing and counseling at the Institute of Human Genetics of the Ludwig Maximilians University (LMU) were observed at the outpatient clinic of the Department of Dermatology and Allergy of the LMU Hospital in Munich, Germany between July 2020 and August 2025 (Table 1). Eligible patients were included if they had clinically and genetically established BHDS and presented with cutaneous lesions clinically compatible with fibrofolliculomas that were suitable for non‐invasive imaging.

**TABLE 1:** Demographic and genetic data of the patients, Transcript‐ID NM_144997.5 (GRCh37).

Patient	Sex	Age (years)	Nucleotide change	Amino acid change
1	f	64	c.779G > A	p.(Trp260Ter)
2	m	69	c.393T > A	p.(Cys131Ter)
3	m	29	c.1177‐5_1177‐3del	p.?
4	f	55	c.251_264del	p.(Ser87AlafsTer8)
5	f	72	c.544_552dup	p.(Leu182_Asn184dup)
6	f	37	c.1285dup	p.(His429ProfsTer27)
7	m	38	c.1300G > C	p.(Glu434Gln)
8	f	45	c.1285dup	p.(His429ProfsTer27)
9	m	82	c.779G > A	p.(Trp260Ter)
10	m	38	c.1062 + 1G > A	p.?
11	f	28	c.1285dup	p.(His429ProfsTer27)
12	m	69	c.573del	p.(Lys192ArgfsTer31)
13	f	49	c.605del	p.(Gly202AlafsTer21)
14	m	69	c.887C > G	p.(Ser296Ter)
15	m	60	c.1285dup	p.(His429ProfsTer27)

Following dermoscopy, suspected skin lesions were imaged using RCM and LC‐OCT to systematically analyse the morphologic and microstructural features of fibrofolliculomas.

RCM was conducted using the VivaScope 1500 static and 3000 handheld device (Vivacope GmbH, Munich, Germany) with 830 nm diode laser in reflection mode. Clinically suspicious areas were scanned horizontally into the depth by using the “VivaStacks” function as an optical biopsy [16]. LC‐OCT was conducted with the deepLive System (DAMAE, Paris, France) [15]. Each lesion was imaged using the vertical (en‐coupe), horizontal (en‐face) and 3D modalities of the device. In selected cases, representative imaged lesions were biopsied to enable direct imaging–histology correlation.

All images were analyzed by a board‐certified dermatologist and specialist in non‐invasive imaging and dermatopathology (MD). Unclear cases were reviewed by a second senior dermatologist who is also specialized in non‐invasive imaging (ES).

According to the gold‐standard histopathology the following morphological features were evaluated:

- Centrally located, dilated follicular unit, with or without keratinous debris or hair shaft.
- Branching chords and strands of epithelial cells radiating from a follicular structure with infundibular features.
- Strands may anastomose or rejoin the infundibulum at several points.

One representative lesion per patient was included in the analysis (*N* = 15 lesions from 15 patients) to avoid clustering bias. The selected lesion was clinically representative of the patient's typical BHDS‐associated papules and was chosen based on accessibility for imaging, adequate image quality, and absence of confounding features such as excoriation, inflammation or prior destructive treatment.

Descriptive statistics were used to summarize patient characteristics and imaging findings. Categorical variables are presented as absolute numbers and percentages. Given the single‐arm observational design without a control group, no inferential statistical testing was performed for the prevalence of phenotypic or imaging features. Accordingly, p‐values were not calculated or reported. Additionally due to the exploratory nature and limited sample size, formal blinding and interobserver agreement analysis were not performed.

The study was conducted according to the principles of the Declaration of Helsinki and international guidelines concerning human studies. The study was approved by the local ethics committee of LMU, approval number 17–699 on 24. June 2019. All participants provided written informed consent to participate in the study and for the publication of clinical and imaging data, including identifiable clinical photographs where applicable. The data supporting the findings of this study are available from the corresponding author upon reasonable request due to ethical restrictions and protection of patient privacy.

## 3 Results

Among the 15 enrolled patients diagnosed with BHDS, the mean age was 53,6 years (age‐range 29 ‐ 82 years), with a slight female predominance comprising 53% of the cohort.

**FIGURE 1:**
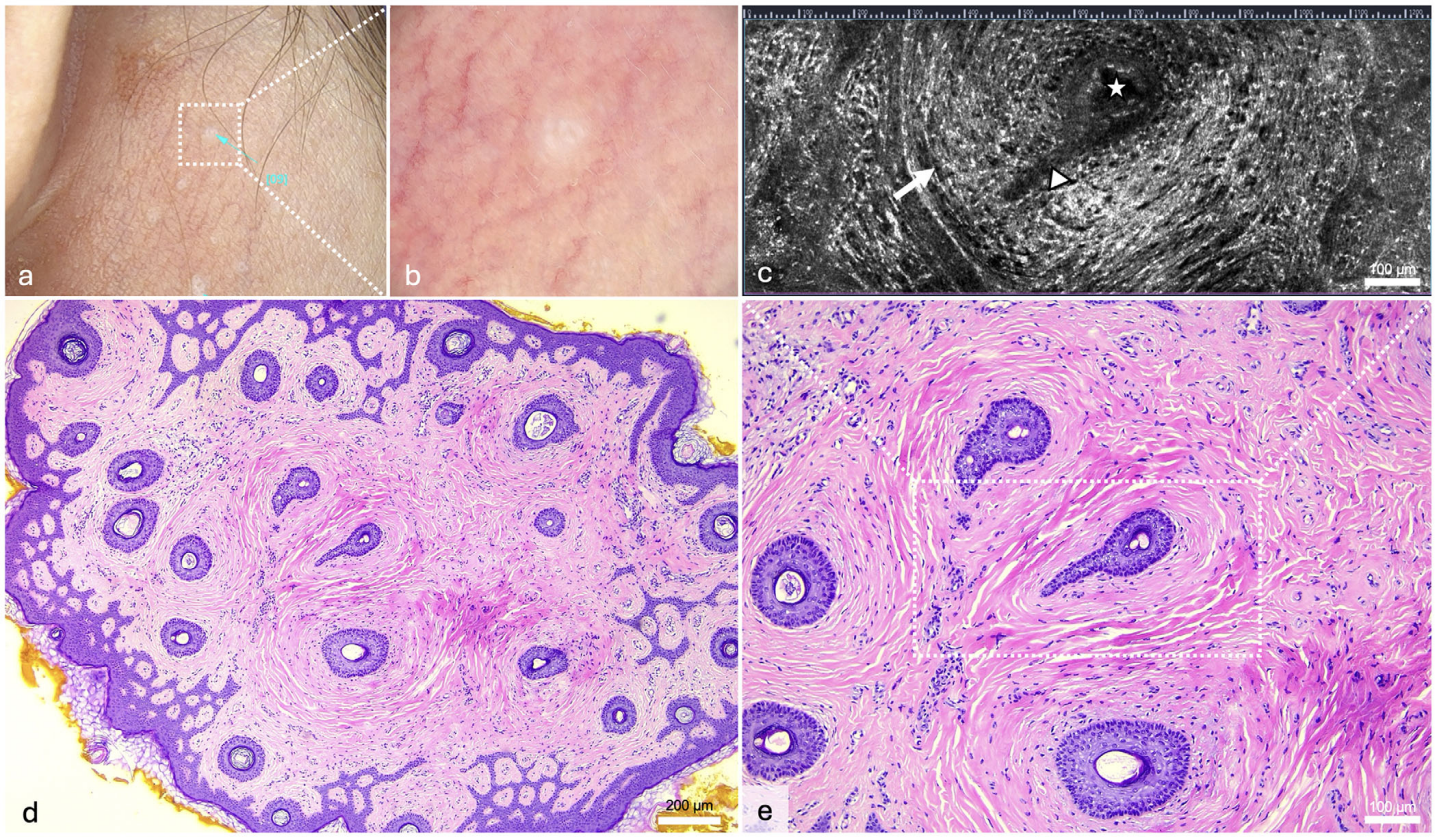
Fibrofolliculoma in a patient with BHDS. (a) Clinical image of the retroauricular region and (b) the dermoscopic image (x10). (c) Corresponding LC‐OCT image (c) with a dilated follicular unit (white star) and rejoining epithelial strand (white triangle) surrounded by onion‐like shaped hyperreflective bundles (arrow). (d) Histology: Hematoxylin‐eosin‐stained sections: Fibrofolliculoma horizontally sliced showing typical strands of epithelial cells radiating from a centrally located follicular structure in overview (d,50x) and (e) detail (100x).

**FIGURE 2:**
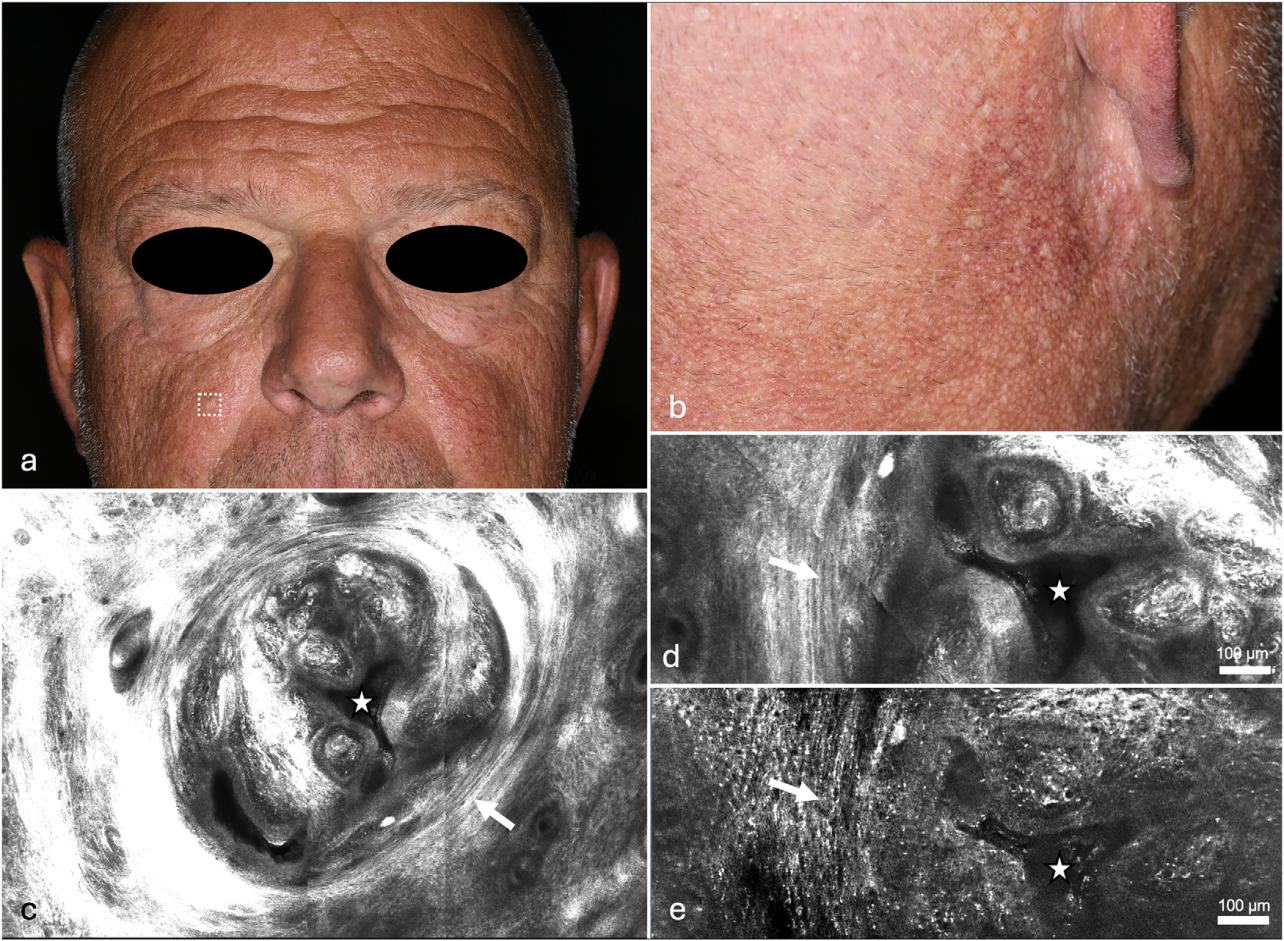
Characteristic features in patients with BHDS. (a) Clinical images showing a BHDS patient with fibrofolliculomas on the face, (b) retroauricular region and the upper neck, (c) In‐vivo discrimination of fibrofolliculomas in horizontal “en‐face” RCM overview “VivaBlock” images using the VivaScope 1500 device, (d) Corresponding high magnification detailed image and (e) DAMAE deepLive LC‐OCT device. A well‐demarcated hyporeflective follicular unit (white star) is surrounded by onion‐like shaped radial hyperreflective bundles of epithelial strands (white arrows), consistent with their histopathological features.

RCM and LC‐OCT images revealed distinct microstructural features of fibrofolliculomas: In RCM a dilated follicular unit was seen in 12/15 cases (80%, Figure 2d), while in LC‐OCT this feature was seen in 13/15 (87%, Figure 1c). The characteristic round, onion‐like shaped hyperreflective structures with a well‐demarcated perifollicular halo, corresponding to the histopathological features of branching chords and strands of epithelial cells was seen in all cases (RCM and LC‐OCT 100%). Rejoining strands were visible in 11/15 RCM cases (73%) and 12/15 LC‐OCT cases (80%).

While both RCM and LC‐OCT provided high‐resolution visualization of cellular and architectural features, LC‐OCT proved superior for characterizing fibrofolliculomas due to its greater penetration depth. This allowed detailed assessment of the perifollicular stroma, epithelial cords and deeper follicular structures, which are often only partially visible or not accessible with RCM because of its limited imaging depth.

## 4 Discussion

In the context of BHDS, the clinical presentation of cutaneous fibrofolliculomas often shares similarities with several other dermatological conditions, including milia, papules and other benign or even malignant skin disorders [2]. Diagnosis remains challenging due to overlapping clinical features and histopathological characteristics, necessitating a comprehensive approach [13].

RCM and LC‐OCT imaging can aid in discerning the underlying structural differences, with fibrofolliculomas displaying distinctive perifollicular halos and characteristic microstructural patterns not typically observed in other benign skin disorders [15, 16]. Moreover, the absence of inflammatory infiltrates and the presence of onion‐like fibrous stroma in fibrofolliculomas serve as key discriminators, as highlighted by our imaging findings. This comprehensive imaging approach, coupled with clinical correlation, contributes significantly to the accurate discrimination of fibrofolliculomas in BHDS.

The application of RCM and especially LC‐OCT in our study demonstrates their potential as valuable adjuncts in the diagnosis of fibrofolliculomas, aiding clinicians in overcoming the diagnostic challenges associated with the clinical resemblance to other cutaneous lesions. The ability of these imaging modalities to provide real‐time visualization of tissue microstructures and cellular details augments our understanding of the distinctive optical and microstructural features of fibrofolliculomas, underscoring their clinical relevance in facilitating precise and targeted diagnostic approaches for BHDS‐related dermatological conditions.

From a practical clinical perspective, patients with suspected BHDS‐associated fibrofolliculomas should therefore undergo a structured diagnostic assessment integrating clinical morphology, personal and family history, dermoscopy, and, where available, non‐invasive imaging.

In patients presenting with multiple, dome‐shaped, whitish or skin‐colored facial papules, particularly in the setting of pulmonary cysts, spontaneous pneumothorax, renal tumors, or a positive family history, BHDS should be considered and genetic evaluation for FLCN variants should be initiated. Dermoscopy may serve as an accessible first‐line tool to support lesion characterization and guide selection of representative lesions for further evaluation. In specialized centers, RCM and LC‐OCT may provide additional real‐time architectural and cellular information, helping to differentiate fibrofolliculomas from clinically overlapping adnexal or follicular lesions and to select lesions requiring biopsy or treatment. However, histopathological confirmation remains essential in atypical cases, solitary lesions, diagnostically uncertain presentations, or before destructive lesion‐directed therapies when diagnostic confidence is insufficient.

This stepwise approach is consistent with recent efforts to integrate dermoscopy and LC‐OCT into diagnostic–therapeutic algorithms for adnexal tumors. For example, Di Guardo et al. recently proposed a diagnostic–therapeutic flowchart for solitary trichoepithelioma incorporating dermoscopy and LC‐OCT as complementary tools in clinical decision‐making. [17] A comparable framework may be useful in BHDS‐related dermatological care, particularly for distinguishing fibrofolliculomas from phenotypically similar adnexal neoplasms and for rationally selecting lesions for biopsy, follow‐up, or treatment.

A limitation of our study is the relatively small sample size of 15 patients. However, given the rarity of BHDS with an estimated prevalence of fewer than 1 in 200,000 individuals [9] and its underdiagnosis due to clinical overlap with common conditions such as acne, rosacea, or sebaceous hyperplasia, this cohort represents a considerable number of systematically investigated cases. Nevertheless, the small sample size limits statistical power and generalizability. Second, this was an exploratory, single‐arm observational imaging study without a control cohort of clinically similar non‐BHDS lesions. Therefore, diagnostic specificity, discriminatory performance, sensitivity, and specificity of the proposed imaging features could not be determined. As image interpretation was conducted in the context of known BHDS diagnoses, observer bias cannot be excluded, and agreement metrics such as kappa values were not available. Finally, access to LC‐OCT and CLSM remains limited and may not be available in all dermatology centers, and image acquisition and interpretation require dedicated equipment, time, and specific expertise. Larger, controlled, multicenter studies including clinically relevant comparator lesions, blinded independent readers, standardized imaging protocols, systematic histological correlation, and formal diagnostic accuracy analyses are therefore required to validate these preliminary findings and to further refine imaging criteria for fibrofolliculomas and their differential diagnoses.

## 5 Conclusion

The successful visualization of BHDS‐related fibrofolliculomas underscores the clinical utility of RCM and LC‐OCT as valuable adjuncts in the diagnostic armamentarium for cutaneous lesions. The ability of these imaging modalities to provide high‐resolution, real‐time visualization of tissue architecture and cellular morphology may not only improve diagnostic precision but also support therapeutic planning and patient management by helping to confirm suspicious lesions before established lesion‐directed treatments, such as biopsy, curettage, or laser‐based approaches, are considered.

## Funding

The authors declare that they received no funding.

## Conflicts of Interest

The authors declare no conflict of interest.

## Ethics Statement

The study was approved by the local ethics committee of the Ludwig‐Maximilians University Munich (Ref‐No.17‐699).

## Data Availability

The data that support the findings of this study are available on request from the corresponding author. The data are not publicly available due to privacy or ethical restrictions.
